# Quantifying the Anthropogenic Footprint in Eastern China

**DOI:** 10.1038/srep24337

**Published:** 2016-04-12

**Authors:** Chunlei Meng, Youjun Dou

**Affiliations:** 1 Institute of Urban Meteorology, China Meteorological Administration, Beijing, 100089, China; 2 State Key Laboratory of Severe Weather, Chinese Academy of Meteorological Sciences, Beijing, 100081, China

## Abstract

Urban heat island (UHI) is one of the most focuses in urban climate study. The parameterization of the anthropogenic heat (AH) is crucial important in UHI study, but universal method to parameterize the spatial pattern of the AH is lacking now. This paper uses the NOAA DMSP/OLS nighttime light data to parameterize the spatial pattern of the AH. Two experiments were designed and performed to quantify the influences of the AH to land surface temperature (LST) in eastern China and 24 big cities. The annual mean heating caused by AH is up to 1 K in eastern China. This paper uses the relative LST differences rather than the absolute LST differences between the control run and contrast run of common land model (CoLM) to find the drivers. The heating effect of the anthropogenic footprint has less influence on relatively warm and wet cities.

China now is experiencing an unprecedented urbanization movement^1^. Rapid urbanization process brings unpredictable influences on urban climate and ecosystem^2^. Methods to quantify the anthropogenic footprints are crucial important in urban climate and ecology study. Urban heat island (UHI) and surface urban heat island (SUHI)^3^,^4^,^5^ are research hotspots in urban climate study, but quantitative separation the contributions of land use and land cover change (LULCC), greenhouse gases emission, and anthropogenic heat (AH)^6^,^7^,^8^,^9^ is still a big problem.

Many methods^10^,^11^,^12^ used to parameterize the spatiotemporal pattern of AH, but most of them are based on Sailor and Lu^13^, namely each profile consists of heat released from the following three components: the building sector, the transportation sector, and human metabolism, which are very complicated and not universal.

Nighttime light data provided by the Defense Meteorological Satellite Program’s Operational Linescan System of NOAA National Geophysical Data Center (NOAA DMSP/OLS) has been widely used in urban study, such as urban footprint boundary^14^, urban impervious surfaces^15^, urban dynamic development^16^,^17^, gross domestic product (GDP)^18^, disaster^19^, light pollution^20^,^21^, population^22^, economic activity^23^, and poverty assessment^24^ etc.

In this paper, the AH is considered as the radiation source term in surface energy balance equation, which is generated by humans and human activity; this is demonstrated by many documents^25^,^26^,^27^,^28^,^29^,^30^. DMSP/OLS nighttime light data reflects the visible and near-infrared radiance at night, which is proportional to the radiation energy at night, so it is also proportional to the AH at night. Based on the discussion above, this paper uses DMSP/OLS data to parameterize the spatial pattern of AH in eastern China and 24 big cities, this method is universal and superior to the method based on Sailor and Lu for the following reasons. Firstly, the nighttime light data is worldwide, so we can obtain the global spatial pattern of the AH at the same time. Secondly, DMSP/OLS data comprises two aspects of information, which is population density and economic development, so it is an ideal alternative data to parameterize the AH^3^. Finally, this paper developed a very simple but robust method to retrieve the AH, so the complicated work which is necessary in the method based on Sailor and Lu such as data exploring and data calculating are all not needed.

The temporal pattern of the AH is based on the diurnal profile of AH used in Weather Research and Forecasting (WRF) Model^31^. Two experiments were designed and performed to quantify the influences of the AH to land surface temperature (LST) in eastern China and 24 big cities. In order to find the climate factors which influence the heating effect of the AH, in this paper, we use the relative LST differences rather than the absolute LST differences between these two experiments to find the drivers. The relative LST differences are defined as the absolute annual average land surface temperature differences divided by the AH.

## Data and Method

### Satellite Data

**Figure 1:**
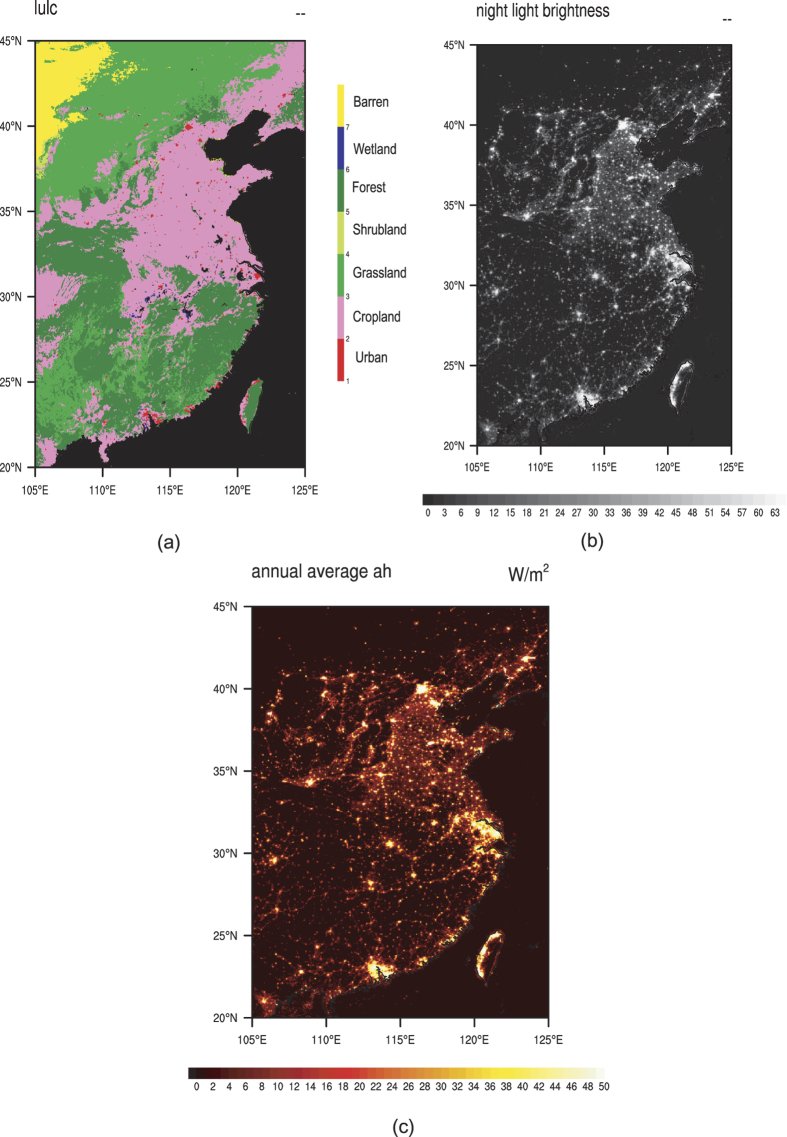
(**a**) MODIS LULC in eastern China; (**b**) DMSP/OLS nighttime light data in eastern China; (**c**) Annual average of the AH in eastern China. (Generated by NCAR Command Language (NCL) Version 6.3.0, ref. 38).

Land cover and land use (LULC) data is from MODIS. We used 0.05^o^ spatial resolution LULC data (MCD12C1) in 2012, downloaded from the website of https://lpdaac.usgs.gov/dataset_discovery/modis/modis_products_table/mcd12c1. The LULC data is transformed from IGBP classification to USGS classification (Fig. 1a). The open shrublands and closed shrublands are both considered as shrubland. The woody savannas and savannas are both considered as savanna. The permanent wetland is considered as wooded wetland. The cropland and natural vegetation mosaic is considered as cropland and woodland mosaic.

Nighttime light data is from NOAA DMSP/OLS in 2012 (Fig. 1b), downloaded from the website of http://ngdc.noaa.gov/eog/dmsp/downloadV4composites.html. These image products provide gridded cell based annual cloud-free composited stable nighttime lights with a digital number (DN) ranged from 0 to 63. The DMSP/OLS nighttime light data was upscaled from 30 seconds spatial resolution to 0.05^o^. The DMSP/OLS image has good correlation with the LULC data, in the urban area, the DN is relatively big.

### Meteorological Forcing Data

We used the meteorological forcing data originated from Global Land Data Assimilation System (GLDAS)^32^ to drive the land surface model. The GLDAS data was interpolated spatially and temporally from 0.25^o^ and 3 hr to 0.05^o^ and 1 hr respectively in order to meet the needs of the study. These data include near surface air temperature, near surface air humidity, wind speed, wind direction, near surface air pressure, precipitation, downward solar radiation and downward longwave radiation.

### Statistical Data

A generalized approach for estimating diurnal profiles of AH for cities has been previously presented^13^. The diurnal profiles data was used in weather research and forecasting model (WRF)^31^. A statistical diurnal profiles data in downtown Beijing (DN = 63) is used in this paper^33^. We use the DMSP/OLS nighttime light data to expand this data to eastern China.

### Land Surface Model

We used the common land model (CoLM)^34^ to simulate the land surface temperature (LST). Two experiments were designed and performed to quantify the influences of AH to LST in eastern China and 24 big cities. For the control run, AH is not added in the land surface energy balance equation; while for the contrast run, AH is added in the land surface energy balance equation as the radiation source.

### Anthropogenic Heat Parameterization

We used a very simple but robust method to parameterize the spatial pattern of the anthropogenic heat in eastern China. Here, we assumed the AH release is proportional to the nighttime light^35^,^36^. Using this method, the annual average of the AH is calculated as follows (Fig. 1c):
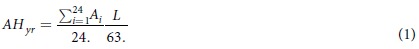


where *AH*_*yr*_ is the annual average AH; *A*_*i*_ is the AH in *i* o’clock which is calculated based on the statistical data; *L* is nighttime light brightness which is from 0 to 63.

## Results and Discussions

Before discussing the results, first we described two important terms used in this paper. The absolute annual average LST differences is the contrast run annual average LSTs subtract the control run annual average LSTs; the relative annual average LST differences is the absolute annual average LST differences divided by the AH.

**Figure 2:**
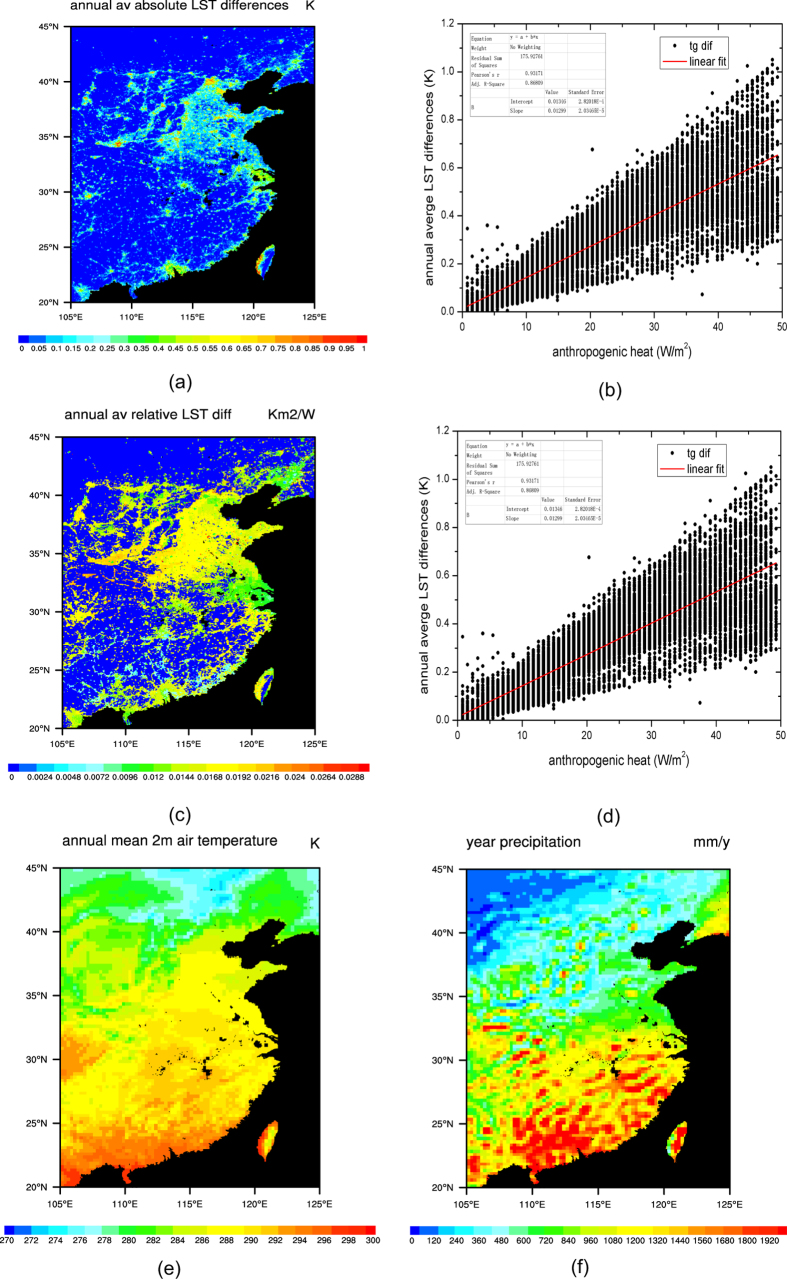
(**a**,**c**) Absolute and relative annual average LST differences between the control run and the contrast run in eastern China; (**b**,**d**) Scatter plot of the AH and the absolute and relative annual average LST differences; (**e**) annual mean m temperature in eastern China; (**f**) annual aggregate precipitation in eastern China. (Figures (**a**,**c**,**e**,**f**) are generated by NCAR Command Language (NCL) Version 6.3.0, ref. 38; Figures (**c**,**d**) are generated by Origin 9.0, http://originlab.com/).

We run the CoLM in a whole year of 2013. We compared the absolute (Fig. 2a) and relative (Fig. 2c) annual average LST differences between the control run and the contrast run in eastern China; and find their relationships with the AH (Fig. 2b,d) respectively. After introducing the AH, the annual average LST increased up to 1 K. The absolute annual average LST differences are basically proportional to the AH. The relative annual average LST differences are weakly inversely proportional to the AH. We compared the spatial pattern of the relative annual average LST differences with the local climate background, e.g. the annual average solar radiation, annual average longwave radiation, annual average wind speed, annual average near surface air pressure, annual average 2 m air temperature, annual average 2 m air relative humidity, and annual aggregate precipitation, and found they are associated with the annual mean 2 m air temperature (Fig. 2e) and annual aggregate precipitation (Fig. 2f). In northern and western research regions, where the climate is cold and dry, the relative annual average LST differences are relatively large; while in the southern and eastern research regions, where the climate is warm and wet, they are relatively small.

**Figure 3:**
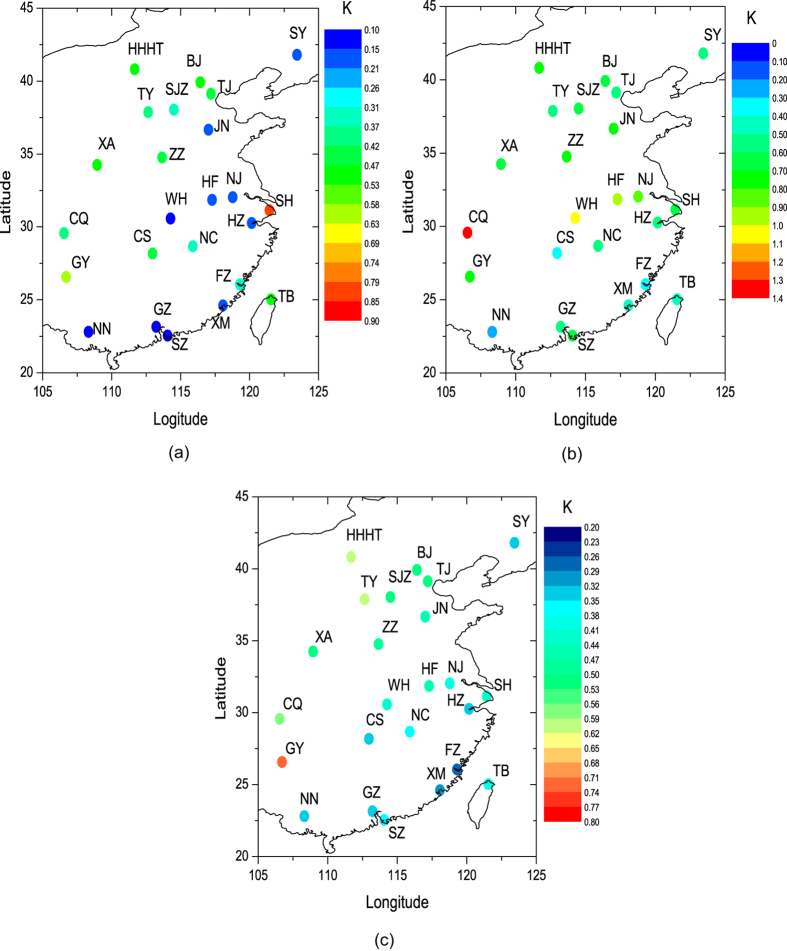
Absolute LST differences between the control run and the contrast run in 24 big cities in eastern China. (**a**) Monthly average in July; (**b**) Monthly average in December; (**c**) Annual average. BJ: Beijing; TJ: Tianjin; SJZ: Shijiazhuang; TY: Taiyuan; HHHT: Huhehaote; SY: Shenyang; JN: Jinan; NJ: Nanjing; SH: Shanghai; HZ: Hangzhou; HF: Hefei; FZ: Fuzhou; XM: Xiamen; NC: Nanchang; ZZ: Zhengzhou; WH: Wuhan; CS: Changsha; GZ: Guangzhou; SZ: Shenzhen; NN: Nanning; CQ: Chongqing; GY: Guiyang; XA: Xian; TB: Taipei. (Generated by Origin 9.0, http://originlab.com/; Maps are generated by Origin 9.0 too, data are from MICAPS (Meteorological Information Comprehensive Analysis and Process System) version 4.0, http://www.cma.gov.cn/en2014/meteorologicalinstruments/News/201501/t20150107_271396.html, which is widely and freely used in meteorological departments in China and was developed by CMA (China Meteorological Administration).

To confirm this, we compared the LST differences between the control run and the contrast run in 24 big cities in eastern China (Fig. 3a–c); the annual mean AHs of these cities are shown in Table 1. In July, the LST increased from 0.1 to 0.9 K caused by the AH. Cities in northern and western regions, the LST increased relatively large except Shanghai. In December, the LST increased from 0.1 to 1.4 K caused by the AH. Cities in northern and western regions, the LST increased relatively large except Chongqing. For the annual average, the LST increased from 0.2 to 0.8 K caused by the AH. Cities in northern and western regions, the LST increased relatively large except Guiyang.

**Figure 4:**
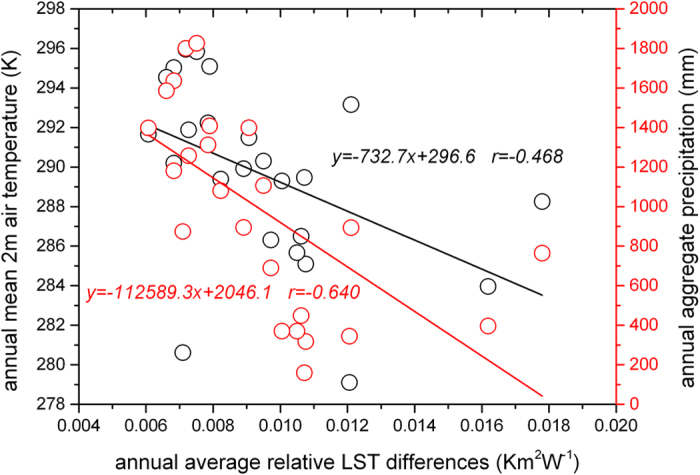
Relationships between the relative LST differences in 24 big cities in eastern China with annual mean 2 m air temperature and annual aggregate precipitation. (Generated by Origin 9.0 http://originlab.com/).

Because the AH for each city is not the same, we use the relative annual LST differences rather than the absolute annual LST differences to find the drivers. We analyzed the relationship between the relative annual LST differences in these 24 big cities with the local background climate^37^ of each city, such as the annual average solar radiation, annual average longwave radiation, annual average wind speed, annual average near surface air pressure, annual average 2 m air temperature, annual average 2 m air relative humidity, and annual aggregate precipitation. After calculating the correlation coefficients between the relative annual LST differences and all the local background climate factors, we found that the main drivers caused the spatial variation of heating are annual mean 2 m air temperature and annual aggregate precipitation (Fig. 4). From Fig. 4, we also can see that the annual mean relative LST differences have more strong correlation with the annual aggregate precipitation. The annual mean 2 m air temperature has positive correlation with the annual mean net radiation. For the cities with relatively large annual mean net radiation or high annual mean 2 m air temperature, the AH occupies relative small part in land surface energy balance, so the variation of LST influenced by AH is also relatively small. For cities with relatively heavy annual aggregate precipitation, the land surface is cooled by evaporation, and for the water-logging land surface, the LST is replaced by water surface temperature, and the variation of LST influenced by AH is weakened. In one word, the heating effect of the anthropogenic footprint has less influence on relatively warm and wet cities.

## Summary

In this paper, we used the DMSP/OLS nighttime light data to parameterize the spatial pattern of the AH. We compared the LST simulation results with and without introducing the AH in eastern China. The results show that the annual average LST in eastern China is increased up to 1 K after introducing the AH. After comparing the relative and absolute differences of LST before and after introducing the AH in 24 big cities in China, we found that the relative annual average LST differences are associated with the local climate background of each city. The heating effect of the anthropogenic footprint has less influence on relatively warm and wet cities.

In the near future, the simulated results of LST will compared with MODIS observation. The spatial pattern of the AH will be extended globally, and the diurnal and seasonal variation of the AH will be reparameterized too. New methods should also be developed to expand the AH spatially in the daytime.

## Additional Information

**How to cite this article**: Meng, C. and Dou, Y. Quantifying the Anthropogenic Footprint in Eastern China. *Sci. Rep.* **6**, 24337; doi: 10.1038/srep24337 (2016).

## Figures and Tables

**Table 1:** Annual average AH in 24 big cities in eastern China.

City name	Annual average AH (W/m^2^)
Beijing	49.25
Shanghai	46.90
Tianjin	49.25
Guangzhou	48.47
Shenzhen	47.68
Taipei	49.25
Nanning	49.25
Guiyang	41.43
Changsha	44.56
Nanchang	46.90
Fuzhou	47.68
Xiamen	46.90
Hangzhou	48.47
Nanjing	47.68
Hefei	46.90
Wuhan	47.68
Chongqing	48.47
Zhengzhou	49.25
Jinan	46.12
Xian	49.25
Shijiazhuang	48.47
Taiyuan	36.74
Hohhot	49.25
Shenyang	49.25
